# Systemic therapy of Cushing’s syndrome

**DOI:** 10.1186/s13023-014-0122-8

**Published:** 2014-08-05

**Authors:** Niels Eckstein, Bodo Haas, Moritz David Sebastian Hass, Vladlena Pfeifer

**Affiliations:** 1 Federal Institute of Drugs and Medical Devices, Kurt-Georg-Kiesinger-Allee 3, Bonn, 53175, Germany; 2 Applied Pharmacy, University of Applied Sciences Kaiserslautern, Campus Pirmasens, Carl-Schurz-Str. 10 – 16, Pirmasens, 66953, Germany

**Keywords:** Cushing’s disease (CD), Cushing’s syndrome (CS), Medicinal therapy, Orphan drug status

## Abstract

Cushing’s disease (CD) in a stricter sense derives from pathologic adrenocorticotropic hormone (ACTH) secretion usually triggered by micro- or macroadenoma of the pituitary gland. It is, thus, a form of secondary hypercortisolism. In contrast, Cushing’s syndrome (CS) describes the complexity of clinical consequences triggered by excessive cortisol blood levels over extended periods of time irrespective of their origin. CS is a rare disease according to the European orphan regulation affecting not more than 5/10,000 persons in Europe. CD most commonly affects adults aged 20–50 years with a marked female preponderance (1:5 ratio of male vs. female). Patient presentation and clinical symptoms substantially vary depending on duration and plasma levels of cortisol. In 80% of cases CS is ACTH-dependent and in 20% of cases it is ACTH-independent, respectively. Endogenous CS usually is a result of a pituitary tumor. Clinical manifestation of CS, apart from corticotropin-releasing hormone (CRH-), ACTH-, and cortisol-producing (malign and benign) tumors may also be by exogenous glucocorticoid intake. Diagnosis of hypercortisolism (irrespective of its origin) comprises the following: Complete blood count including serum electrolytes, blood sugar etc., urinary free cortisol (UFC) from 24 h-urine sampling and circadian profile of plasma cortisol, plasma ACTH, dehydroepiandrosterone, testosterone itself, and urine steroid profile, Low-Dose-Dexamethasone-Test, High-Dose-Dexamethasone-Test, after endocrine diagnostic tests: magnetic resonance imaging (MRI), ultra-sound, computer tomography (CT) and other localization diagnostics. First-line therapy is trans-sphenoidal surgery (TSS) of the pituitary adenoma (in case of ACTH-producing tumors). In patients not amenable for surgery radiotherapy remains an option. Pharmacological therapy applies when these two options are not amenable or refused. In cases when pharmacological therapy becomes necessary, Pasireotide should be used in first-line in CD. CS patients are at an overall 4-fold higher mortality rate than age- and gender-matched subjects in the general population. The following article describes the most prominent substances used for clinical management of CS and gives a systematic overview of safety profiles, pharmacokinetic (PK)-parameters, and regulatory framework.

## Introduction

**Figure 1:**
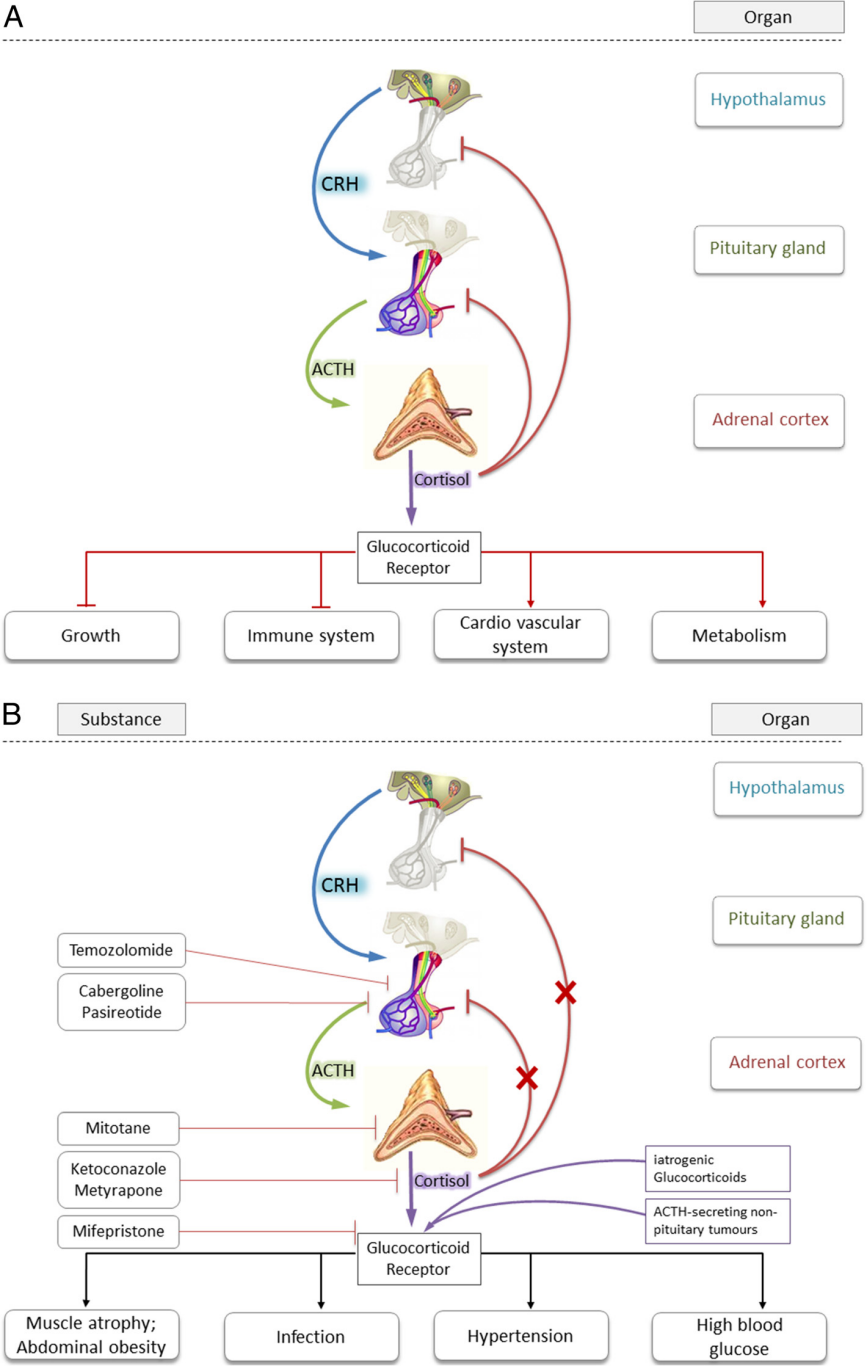
**Schematic overview of the hypothalamic-pituitary-adrenal (HPA) axis. A)** Schematic overview of the hypothalamic-pituitary-adrenal (HPA) axis. Depicted is the physiological hierarchical pathway to cortisol-secretion from the *zona fasciculata* of the adrenal cortex including negative feed-back loop. Also shown are the most prominent effects of cortisol. **B)** Schematic overview of HPA axis in pathologically de-regulated CS-patients. Pathological condition may lead to CRH, ACTH, and cortisol overproduction and impaired negative feed-back loop. Also shown are most hazardous clinical side-effects of hypercortisolism and target of cortisol blockade.

Both, Cushing’s disease (CD) and Cushing’s syndrome (CS), are rare diseases characterized by high cortisol blood levels and impairment of circadian oscillation [1]. CD derives from pathologic adrenocorticotropic hormone (ACTH) secretion usually triggered by micro- or macroadenoma of the pituitary gland [2]. It is, thus, a form of secondary hypercortisolism. In contrast, CS describes the complexity of clinical consequences triggered by excessive cortisol blood levels over extended periods of time irrespective of their origin [3]. Clinical manifestation of CS, apart from corticotropin-releasing hormone (CRH-), ACTH-, and cortisol-producing (malign and benign) tumors may also be by exogenous glucocorticoid intake (Figure 1B). Patient presentation and clinical symptoms substantially vary depending on duration and plasma levels of cortisol. In 80% of cases CS is ACTH-dependent and in 20% of cases is ACTH-independent, respectively [4]. Clinical differentiation with respect to the onset of a certain treatment algorithm is by ACTH dependency and independency, respectively:

 ACTH-dependent

 ➢ Pituitary adenoma (CD in strict sense) ~ 70% [5]

 ➢ Ectopic secretion of ACTH by non-pituitary tumors ~ 15% (i.e. neuroendocrine tumors such as small-cell lung cancer (SCLC), carcinoid tumors, and medullary carcinoma of the thyroid) [6]

 ➢ Ectopic secretion of CRH by non-hypothalamic tumors causing pituitary hypersecretion of ACTH < 1% [7]

 ➢ Iatrogenic or factitious CS due to administration of exogenous ACTH < 1%

 ACTH-independent

 ➢ Adrenocortical adenomas and carcinomas ~ 20% [8]

 ➢ Primary pigmented nodular adrenocortical disease < 1% [9]

 ➢ Bilateral ACTH-independent adrenal hyperplasia < 1% [10]

**Figure 2:**
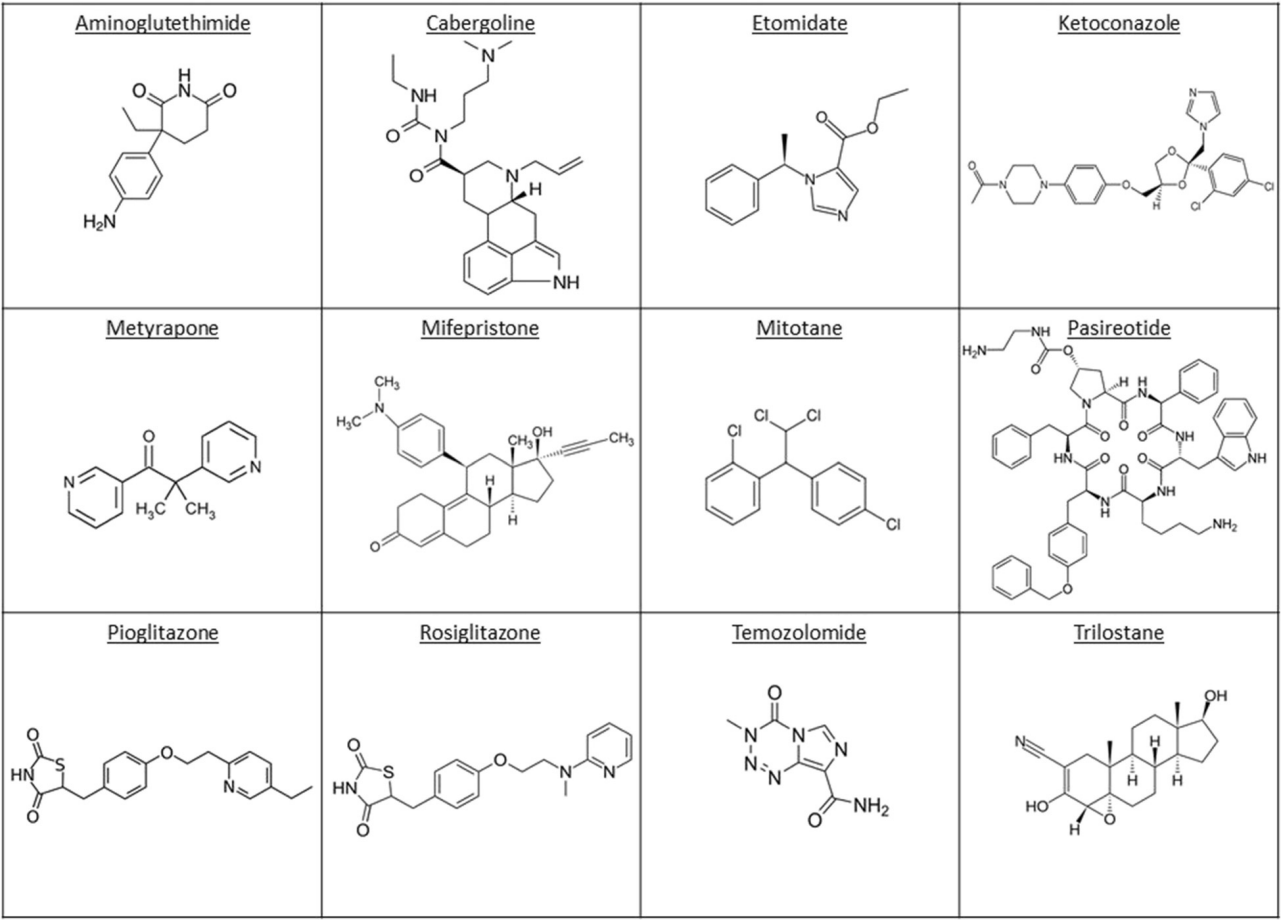
Chemical structures of medicinal products used for systemic treatment of CS in alphabetical order.

However, systemic treatment options are limited and clinical evidence of these options is scarce (with Pasireotide as the exception from the rule). The regulatory status of pharmacological treatment options are reflected in Table 1. Chemical structures of medicines used in-label and off-label are depicted in Figure 2. According to the European Community Register [11] (accessed on March 12^th^, 2014) the medicinal products were granted an orphan designation in the context of CS.

**Table 1:** Regulatory status of medicines for the treatment of hypercortisolism in alphabetical order

**Drug substance (trade name)**	**Indication(s)**	**Regulatory Status in Europe**	**ATC-Code**^**1**^	**Galenic formulation**	**Route of administration**
Aminoglutethimide (Orimeten®)	Originally approved with the following wording:	Not approved any more	L02BG01	Tablet	Oral
	Indication: CS				
	In some patients < Orimeten > is indicated for preparation of surgery. A long-term administration is possible in case of inoperable patients or in case of recurrence after incomplete adrenalectomy. CS may be due to				
	- Adrenocortical tumors (adenoma and carcinoma)				
	- ACTH released from ectopic ACTH syndrome				
Cabergoline (Dostinex®)	Parkinson’s disease and hyperprolactinemic disorders	Approved	N04BC06; G02CB03	Tablet	Oral
Etomidate (Hypnomidate®)	Hypnomidate is used for anesthesia	Approved	N01AX07	Solution	Intravenous injection
Ketoconazole (Nizoral®)	Treatment of CS	MAA submitted to the EMA^2^	J02AB02	Tablet	Oral
Metyrapone (Metopirone®)	- As a diagnostic test for ACTH insufficiency and in the differential diagnosis of ACTH-dependent CS.	Licensed in Ireland, UK, France based on national procedures	V04CD01	Capsule	Oral
	- For the management of patients with CS.	Trade name: Metopirone®			
Mifepristone (Corluxin®)	Treatment of CS secondary to ectopic ACTH secretion	Not yet approved in CS^3^	G03XB01	Tablet	Oral
		MAA submitted to EMA			
Mitotane (Lysodren®)	Symptomatic treatment of advanced (non-resectable, metastatic or recurrent) adrenal carcinoma	Licensed via central procedure as orphan drug since 2004	L01XX23	Tablet	Oral
		Trade name: Lysodren® (EMEA/H/C/000521)			
Pasireotide (Signifor®)	Second-line treatment of CD in patients where surgery has failed	Licensed via central procedure as orphan drug since 2012	H01CB05	Solution and powder for solution for injection, respectively	Subcutaneous injection
		Trade name: Signifor®			
Rosiglitazone (Avandia®)	Rosiglitazone is used to treat type 2 diabetes mellitus, particularly those who are overweight. It is used in addition to diet and exercise	Currently suspended for use in the European Union, restricted use in the US	A10BG03	Tablet	Oral
Temozolomide (Temodal®)	Off-label in aggressively growing pituitary macroademomas	Licensed (initially via central procedure) for Glioblastoma multiforme and anaplastic astrocytoma since 1999 (EMEA/H/C/000229)	L01AX03	Capsule	Oral

*Abbreviations:* CD, Cushing’s disease; CS, Cushing’s syndrome; EMA, European Medicines Agency; MAA, marketing authorization application; ^1^If off-label use, ATC-Code from other indication is depicted; ^2^in anti-fungal (Nizoral®) indication marketing authorization is suspended in Europe due to hepatotoxicity; ^3^for abortion, licensing status is varying among European Member States.

### Etiology and symptoms

CS is a rare disease according to the European orphan regulation [12] affecting not more than 5/10,000 persons in Europe. CS is a heterogeneous disorder that arises from multiple causes and has a broad spectrum of eventually fatal co-morbidities such as diabetes and hypertension.

*ERCUSYN* is the synonym for The European Registry on Cushing’s Syndrome. In a startling publication from 2011 Valassi et al. [13] describe the baseline demographic and clinical characteristics from:

1. a prospective cohort of 398 CS patients who were recruited from October 1^st^ 2008 (when the database was opened) to October 31^st^ 2010, and

2. a retrospectively collected cohort of 83 patients diagnosed of CS since January 1^st^ 2000 with yearly updates.

This patient population consisted of:

 ➢ 317 (66%) patients suffering from CD

 ➢ 130 (27%) patients who had adrenal-CS

 ➢ 24 (5%) patients who had ectopic-CS

 ➢ 10 (2%) patients classified as having other forms of CS

CS comprises numerous general and endocrine symptoms and side effects some of which might be entailed with fatal outcome. Excess cortisol levels result in (among others):

 ➢ facial plethora

 ➢ hirsutism

 ➢ gonadal dysfunction

 ➢ menstrual irregularities

 ➢ depression

 ➢ infections due to generalized immune suppression

 ➢ striae

 ➢ vascular fragility

 ➢ hypokalemia

 ➢ osteoporosis and eventually fractures

 ➢ muscle weakness

Thus, adverse events are indistinguishable from long-term (sometimes unavoidable) glucocorticoid therapy. The metabolic consequences of cortisol excess include:

 ➢ weight gain

 ➢ central obesity

 ➢ skin atrophy

 ➢ glucose intolerance entailed by diabetes and insulin resistance

 ➢ dyslipidemia

 ➢ hypertension and

 ➢ clotting disorders (eventually even hypercoagulability [14].

Especially weight gain, diabetes, and hypertension are hazardous for patients due to two reasons:

1) They are (apart from infections) responsible for a 50% mortality within the first 5 years after diagnosis mainly due to cardiovascular events [15].

2) These symptoms usually are diagnosed as metabolic syndrome, thereby delaying time to diagnosis [16].

According to current data time to diagnosis after the first symptoms is 6.0 years in mean [17]. In this regard, un-specificity of symptoms is highly problematic alike the slow-progressive nature of the disease [18]. However, months or even years of un-diagnosed CS may cause irreversible organ damage or be fatal due to late-stage diagnosis of malignant disease (i.e. ectopic ACTH producing SCLC). Recovery from the a.m. co-morbidities occurs, but may be delayed or incomplete. The duration of chronic hypercortisolism may determine reversibility of the co-morbidities associated with CD [19]. In addition, CS side-effects lead to an increased cardiovascular risk, thus, rendering even timely diagnosed CS a life-threatening disease, which is also due to infections: 71.4% of the deaths from CS can be attributed to cardiovascular causes or infection [20]. Standardized mortality ratios (SMR) in Cushing patients throughout the Ntali-study were statistically significantly elevated in the overall analysis (SMR 9.3; 95% CI, 6.2 - 13.4, p < 0.001), as well as in all subgroups of patients under investigation. Patients with ectopic CS had the worst outcome with a probability of only 77.6% to survive the next 5 years. A systematic analysis of mortality studies in patients with CS as a consequence of an adrenal adenoma was undertaken by Graversen and colleagues in 2012 [21]. CD patients with persistent disease after initial surgery had a SMR of 3.73 (95% CI: 2.31 - 6.01), whereas mortality of CD patients with initial remission did not differ significantly from the general population (SMR: 1.23 (95% CI: 0.51 - 2.97)). This study confirms the excess mortality in patients with CD if remission after initial surgery is not achieved.

### Epidemiology of Cushing’s syndrome as a rare disease

Approximately 1% of the population use *exogenous* steroids, of which 70% experience one or more adverse events [22]. In contrast, a national register in Denmark reported an annual incidence for *endogenous* CS of two cases per million people [23]. Endogenous CS is usually (in 70% of cases) a result of a pituitary tumor. In general, CS is rare: the reported incidence of endogenous CS worldwide ranges from 0.7 - 2.4 cases per million per year [24]. CD most commonly affects adults aged 20 - 50 years with a marked female preponderance (1:5 ratio of male vs. female). CS patients are at an overall 4-fold higher mortality rate than age- and gender-matched subjects in the general population [25]. When diagnosed the prevalence of co-morbidities has been reported as follows: 58 – 85% of patients have hypertension, 32 – 41% obesity, 20 – 47% diabetes mellitus, 50 – 81% major depression, 31 – 50% osteoporosis, and 38 – 71% dyslipidemia [19].

### Lines of treatment

Depending on the cause of these excessive cortisol concentrations in the patient’s blood, different treatment modalities are possible in routine patient care. Therapy of choice is trans-sphenoidal surgery (TSS) of the pituitary adenoma (in case of ACTH-producing tumors). In patients not amenable for surgery radiotherapy remains an option. Thus, primary lines of therapy in CD (i.e. not medication associated CS) consists of TSS and pituitary irradiation.

TSS is currently the most frequently recommended treatment except for patients who are poor surgical candidates, have invasive tumors, or who refuse surgery. The surgical effectiveness varies depending on expertise in pituitary surgery and the size and extension of the anatomic mass. Hypopituitarism is common after TSS with a range between 13 and 81% [3]. Early remission in terms of cortisol blood level normalization is achieved regularly (Figure 1A). Patients who do not achieve normalization with surgery require additional treatment, usually with radiotherapy and/or medication. If damage to the surrounding normal pituitary tissue occurs during surgery, the patient may require lifelong pituitary hormone replacement. However, an examination of remission and recurrence rates in long-term follow-up studies reveals that potentially up to 40% to 50% of patients could require additional treatment [26]. Thus, systemic (medicinal) treatment of CS may be more important than traditionally thought (Figure 1B).

Pituitary irradiation is usually reserved for patients who have tumor remaining after surgery, for patients who are poor candidates for surgery, and for patients who do not respond adequately to surgery and/or medication. The main disadvantages of radiotherapy are that i) normalization of ACTH secretion may take extended periods of time (eventually years) to occur demanding for medication while success of radiation is awaited, and ii) that patients may develop generalized anterior pituitary insufficiency [27].

**Figure 3:**
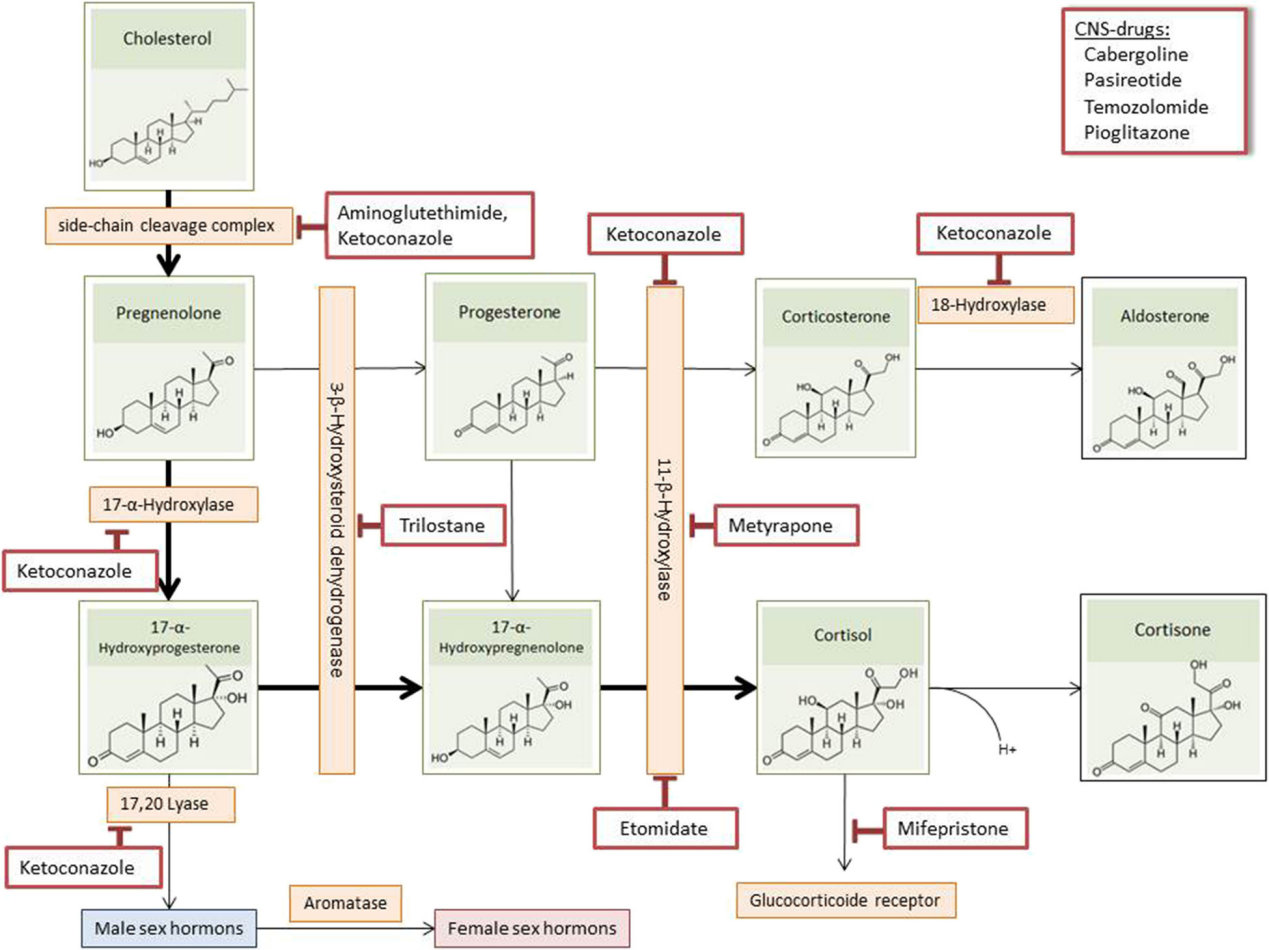
Endogenous biosynthesis and mechanisms of drug inhibition of cortisol.

Pharmacological therapy applies when these two options are not amenable or refused. In cases when pharmacological therapy becomes necessary, Pasireotide should be used in first-line in ACTH-dependent CS. However, systemic pharmacotherapy of CS often shows – sometimes dose-limiting – side effects. A systematic overview of safety profiles is given in Table 2. The source of data in Table 2 is a systematic recherché in section 4.8 of currently (March 2014) approved SmPCs of licensed medicines. In the following sections drugs used in CS treatment (Figure 2) are referred to in alphabetical order (as in all Tables and Figures). Molecular interaction of the respective drug with cortisol-biosynthesis is depicted in Figure 3, whereas PK parameters of the respective substances are listed in Table 3.

**Table 2:** Safety profiles of medicines used for the treatment of hypercortisolism in alphabetical order

	**CNS**	**Nerve disorders**	**Eye disorders**	**Cardiac disorders**	**Lung, airways disorders**	**General disorders and administration site conditions**	**Liver, Bile disorders**	**Endocrine disorders**	**Metabolism and nutrition disorders**	**Genitalia and mammary gland**	**Immune system**
Aminoglutethimide	XX	XX				XX	X	X	X		X
Cabergoline	XX	XX	X	XX	XX	XX					
Etomidate	XX	XX		X	XX	X		X			X
Ketoconazole	X	XX	XX				XX	X		XX	X
Metyrapone		XX						X			
Mifepristone		X				X				XX	
Mitotane	XX	XX	X			XX	XX	XX	XX	XX	
Pasireotide		XX		XX		XX	XX	XX	XX		
Rosiglitazone			XX	XX^1^		XX	X		XX		
Temozolomide	XX	XX	XX	X	XX	XX	XX	X	XX	X	
	**Gastrointestinal disorders**	**Kidney, renal or urinary tract disorders**	**Musculoskeletal and bone disorders**	**Blood and lymphatic system**	**Vascular disorders**	**Skin disorders**	**CMR**	**Infectious and parasitic diseases**	**Psychiatric disorder**	**Ear and labyrinth disorders**	
Aminoglutethimide	XX	X		X	X	XX					
Cabergoline	XX		X	XX	X	XX					
Etomidate	XX		X		XX	XX					
Ketoconazole	XX			X		XX					
Metyrapone	XX	X		X	XX	X			XX		
Mifepristone	XX				X	X		XX			
Mitotane	XX	XX	XX	XX	X	XX	XX	X	XX		
Pasireotide	XX			XX		XX			XX		
Rosiglitazone	XX		XX	XX							
Temozolomide	XX	XX	XX	XX	XX	XX	XX	XX	XX	XX	

*Abbreviations:* XX, common, very common; X, rare, uncommon, not known; CMR, carcinogenic, mutagenic and toxic for reproductive system; ^1^Rosiglitatzone was suspended for use in the European Union due to an increased cardiovascular risk; method of safety profile assessment: between March and June 2014 SmPCs of currently marketed medicinal products were accessed (if not licensed for treatment of CS, then in other indication; i.e. Ketoconazole, Etomidate, Temozolomide, and Rosiglitazone). If not marketed any more (Aminoglutethimide) or suspended (Rosiglitazone) the last approved SmPC was used.

**Table 3:** Clinical pharmacokinetic profiles of medicines used for the treatment of hypercortisolism

	**Aminoglutethimide**	**Cabergoline**	**Etomidate**	**Ketoconazole**	**Metyrapone**
Trade name; sponsoring company	Orimeten; Novartis	Dostinex; Pfizer	Hypnomidate; Janssen-Cilag	Nizoral; Janssen-Cilag	Metopirone; HRA Pharma
European birth date	January 18, 1984	June 24, 2002	October 27, 1978	July 20, 1981	April 1, 1979
Cancer indication	Metastatic breast cancer	No	No	No	No
C_max_ (μg/mL)	5.9^1^	37 ± 8 pg/mL	N/A	3.5	3.7^2^
t_max_ (h)	1.0 - 4.0	0.5 – 4.0	N/A	1.0 - 2.0	1.0
Bioavailability (oral, %)	92 – 98 systemic	N/A^3^[28]	N/A	N/A	N/A
Concomitant food intake effect on bioavailability	With food and water	No significant food effect	N/A	Maximal absorption with food	Recommended with milk or after a meal to minimize nausea and vomiting
Volume of distribution (L/kg) 70-kg subject assumed	N/A	N/A^3^[28]	4.5	N/A	N/A
Major metabolites	N-acetylaminoglutethimide (N-hydroxylaminoglutethimide **if enzyme induction occurs)	6-Allyl-8-β-carboxy-ergoline	N/A	Main metabolism is by oxidation and cleavage of the imidazole- and piperazine-ring systems by hepatic microsomal enzymes.	Metyrapol, an active alcohol metabolite, similar potency in inhibiting adrenal 11-β-hydroxylase
Plasma half-life (h)	12.5 ± 1.6	63 - 69	3 - 5	biphasic 2/8	20 - 26 minutes intravenous, ~2 h oral
Plasma protein binding (%)	21 – 25	41 - 42^4^	76.5	99 (measured in vitro) mainly albumin	N/A
Empirical formula (g/mol)	C_13_H_16_N_2_O_2_ (232.3)	C_26_H_37_N_5_O_2_ (451.6)	C_14_H_16_N_2_O_2_ (244.3)	C_26_H_28_Cl_2_N_4_O_4_ (531.4)	C_14_H_14_N_2_O (226.3)
Mechanism of action	Inhibitor of cytochrome P450 (steroidogenesis and mineralocorticoidgenesis inhibitor)	Dopamine receptor agonist	Inhibits cortisol synthesis by inhibiting CYP11B1, and cytochrome P450scc [29]	Inhibitor of various subtypes of cytochrome P450 (steroidogenesis inhibitor);Inhibitor of side-chain cleavage complex, 17,20-lyase, 11-β-hydroxylase, 18- hydroxylase and 17-α-hydroxylase	Inhibits adreno-corticosteroid synthesis by blocking the 11-β-hydroxylase in the adrenal cortex
Clinical recommended dose	Adrenocortical-adenoma: 2 - 3 × 250 mg adrenocortical-carcinoma: 2 - 4 × 250 mg ectopic ACTH-syndrome: 4 - 7 × 250 mg	Initiation of therapy: 0.25 mg twice a week. Dosage may be increased by 0.25 mg twice a week up to a dosage of 1 mg twice a week according to the patient’s serum prolactin level	Effective hypnotic dose: between 0.15 mg/kg and 0.30 mg/kg; doses of 0.04 – 0.05 mg/kg per h for hypercortisolemia patients [30]	1 × 200 mg q.d.	Daily dose: from 250 mg to 6 g; 6 four-hourly doses of 15 mg/kg, with a minimum dose of 250 mg^5^
Dosage form	Tablet	Tablet	Solution	Tablet	Capsule
Human AUC at the clinical dose	96.8 ng*h/mL	1295 ± 359.3 ng*h/mL [28]	N/A	N/A	N/A
	**Mifepristone**	**Mitotane**	**Pasireotide**	**Temozolomide**	
Trade name; sponsoring company	Corluxin; HRA Pharma	Lysodren; HRA Pharma	Signifor; Novartis	Temodal; Merck Sharp & Dohme	
European birth date	August 25, 1999	April 28, 2004	April 24, 2012	January 26, 1999	
Cancer indication	No	Adrenocortical carcinoma	No	Glioblastoma	
C_max_ (μg/mL)	1.98	13.0^6^[31]	N/A	13.9^7^[32]	
t_max_ (h)	1.0 - 2.0	3.7^6^[31]	0.25 - 0.5	0.5 – 1.5	
Bioavailability (oral, %)	69 absolute by lower dose; higher dose 40	N/A	N/A	Essentially 100^7^[32]	
Concomitant food intake effect on bioavailability	No significant food effect	With food and water	N/A	No information in SmPC	
Volume of distribution (L/kg) 70-kg subject assumed	1.47 ± 0.25 [33]	N/A	Vz/F >100 Liter	0.39^7^[32]	
Major metabolites	RU 42633, RU 42698 and RU 42848	1,1-(o,p’-Dichlorodiphenyl-) ethanoic acid (o,p’-DDA)	N/A	5-Amino-imidazole-4-carboxamide (AIC) and Methyl-hydrazine	
Plasma half-life (h)	90	18 - 159 days	~12	1.8	
Plasma protein binding (%)	98	N/A	88	10 - 20	
Empirical formula (formula weight g/mol)	C_29_H_35_NO_2_ (429.6)	C_14_H_10_Cl_4_ (320.0)	C_59_H_67_N_9_O_9_ (1046.2)	C_6_H_6_N_6_O_2_ (194.2)	
Mechanism of action	Competitive progesterone receptor antagonist	Precise mechanism of action is not known, but an adrenal inhibition is suspected	Binding to four of five somatostatin receptors; (SSTR-1,-2,-3,-5) [34]	After poisoning of TMZ to active metabolite MTIC, alkylation of Guanine-residues at N-7	
Clinical recommended dose	600 mg	Patients are titrated to reach 14 - 20 mg/L plasma concentration starting with 2 - 3 g daily	0.6 mg b.i.d. (0.3 or 0.9 mgdose adaption)^8^	Initially 200 mg/m^2^ daily (pretreated patients: 150 mg/m^2^ daily)	
Dosage form	Tablet	Tablet	Subcutaneous injection	Capsule	
Human AUC at the clinical dose	0.67 ± 0.21 μmol/l*h/mg^9^[35]	N/A	54.1 ± 7.0 ng*h/mL^10^[36]	33.2 μg*h/mL^12^[32]	

*Abbreviations:* AUC, area under the curve; b.i.d., twice daily; C_max_, maximal plasma concentration; q.d., every day; t_max_, time after administration when C_max_ is reached; ^1^500 mg; ^2^750 mg administration; ^3^n = 12, dose = 0.5 mg; ^4^in-vitro study with concentrations of 0.1 - 10 ng/ml; ^5^pediatric population; ^6^measured in dogs, 50 mg/kg, tablets in food; ^7^200 mg/m^2^; ^8^also available as long-acting release formulation in the strengths 20, 40, 60 mg; ^9^AUC_0-inf_; ^10^AUC_0–24 h_: 600 μg q.d. (n = 11); ^11^30 mg single dose in 24 healthy volunteers; ^12^AUC_0–24 h_; source of information: SmPCs of the corresponding medicines unless otherwise indicated (if not licensed for treatment of CS, then in other indication; i.e. Ketoconazole, Etomidate, and Temozolomide). If not marketed any more (Aminoglutethimide) the last approved SmPC was used.

### Aminoglutethimide

Aminoglutethimide from its molecular mechanism is a non-selective, non-steroidal aromatase inhibitor. Due to its non-selective mechanism of action it also inhibits endogenous cortisol synthesis. Schteingart et al. back in 1966 showed that Aminoglutethimide can alleviate clinical symptoms of CS in a patient suffering from metastatic adrenal cancer [37]. However, the same consortium of authors found one year later that its effect can be overruled by excessive secretion of ACTH [38]. Following a report of three patients receiving Aminoglutethimide in combination with Metyrapone, these substances were also given concomitantly [39]. However, Aminoglutethimide is marketed no more.

### Cabergoline

Two types of receptors are widely expressed at the surface of pituitary adenoma cells: the somatostatin receptor subtype 5 (SSTR-5) and the dopamine receptor subtype 2 (D_2_). As mentioned above, SSTR-5 is occupied by Pasireotide prevalently compared to other somatostatins. Dopamine agonists (e.g. bromocriptine, quinagolide, cabergoline) bind to D_2_ receptors in the pituitary gland.

In CD corticotroph adenomas mainly express D_2_ receptors (and SSTR-5). Agonists at these receptors inhibit ACTH-release in cell culture of corticotroph adenomas. In those in vitro model systems compounds that target SSTR-5 (like Pasireotide) or D_2_ (cabergoline) have shown efficacy in subsets of patients in the clinical setting. Combination therapy by administration of both types of compounds yielded promising results.

These clinical reports of enhanced efficacy of combination therapy with SSTR- and dopamine agonist treatment (originally initiated for pituitary adenoma therapy in suppressing growth hormone hypersecretion) lead to the novel concept of somatostatin-dopamine chimeric molecules, e.g. BIM-23A760 [40]. Preliminary in vitro results suggest that the affinity to D_2_ receptors is crucial for inhibition of prolactin gene expression and growth hormone secretion. However, the impact of these substances on CS in patients remains to be elucidated. In addition, a recent study showed that D_2_ receptors are also expressed in the majority of ectopic ACTH-producing syndrome (EAS) cases and that cabergoline may decrease cortisol levels in certain subsets of these patients [41].

### Etomidate

Etomidate (just like Ketoconazole) from the chemical perspective is an imidazole derivative. It was developed in the sixties as parenteral hypnotic drug [42]. Mechanistically, Etomidate is a centrally acting γ-aminobutyric acid type A (GABA-A) receptor agonist [43]. Low serum cortisol levels resulting in hypoadrenalism was a randomly discovered side effect of Etomidate [44]. Etomidate (like Metyrapone) inhibits the mitochondrial cytochrome P450 (CYP)-dependent adrenal enzyme 11-β-hydroxylase that catalyzes the production of cortisol from deoxycortisol, thereby lowering cortisol blood levels within hours [45]. In a recent systematic review, Preda et al. came to the conclusion that Etomidate might be of clinical benefit in last-line therapy under intensive care condition. In the outpatient endocrinology setting for patients with severe hypercortisolism there might be a role for Etomidate [30]. Etomidate usually is thought to be efficacious only in hypnotic doses, but this appears not to be the case: in the Preda publication, non-hypnotic doses of only 0.3 mg/kg were active in lowering cortisol levels.

### Glitazones

Glitazones (Thiazolidinedions) like Rosiglitazone or Pioglitazone are anti-diabetic insulin sensitizing drugs mediating their effects via activation of the transcription factor peroxisome proliferator-activated receptor γ (PPAR γ). Activation of PPARγ results in effects on adipogenesis, carbohydrate and lipid metabolism, inflammation processes, and cell proliferation [46]. Additionally, PPARγ agonists have been shown to inhibit the growth of several tumor cells derived from lung, breast, prostate, and colon cancer tissues [47]–[50]. Immunocytochemical PPARγ expression could be shown in autopsy-derived human pituitary tissue. PPARγ expression was primarily restricted and co-localized with ACTH [51]. However, in pituitary tumors, including ACTH secreting tumors, PPARγ expression is increased as compared to the normal pituitary [52]. In rat and human corticotroph adenoma cell lines Rosiglitazone decreased tumor cell growth, increased apoptosis, and lowered ACTH secretion by inhibiting mRNA expression of its precursor-protein pro-opiomelanocortin (POMC). PPARγ has furthermore been shown to act as tumor suppressor gene in animals [53],[54]. It was hypothesized that glitazones might be useful in treating corticotroph pituitary adenomas by inhibiting ACTH synthesis and secretion and tumor growth. Subsequently, clinical studies investigating the role of glitazones in the treatment of CD were conducted:

In one study, 14 patients with active CD (7 untreated and 7 after unsuccessful surgery) were treated with 8 – 16 mg of Rosiglitazone for 1 – 7 months [55]. In six patients, 24-hour urinary free cortisol (UFC) was significantly lowered and two of them showed clinical improvement at 7-month follow-up. However, after 10 month cortisol levels increased and clinical signs relapsed [56]. Pre-operative Rosiglitazone treatment (8 mg/day) of two patients with pituitary-dependent CS lowered cortisol levels in the 24-hour urine and lead to clinical improvement [57]. In a study of 10 patients, four prior to surgery, four following relapse after surgery, and two immediately after failed surgery treated with 4 – 16 mg of Rosiglitazone for 1 – 8 months, no consistent reductions in UFC levels were found. Only 3 of 10 patients had normalized urinary cortisol levels up to 8 months [58]. Side effects reported included edema, weight gain, somnolence, and increased hirsutism. Although most studies used Rosiglitazone, one study with Pioglitazone is available [59]. In none of the five patients with CD treated with 45 mg Pioglitazone for 30 days any UFC responses were observed. Taken together, glitazone treatment failed to reproduce the effects seen in vitro and in animal studies. Only Rosiglitazone could be shown to be effective at least in a small subset of patients. However, Rosiglitazone is currently suspended in the European Union due to an increased cardiovascular risk.

### Ketoconazole

Ketoconazole originally was developed as an anti-fungal medicine. In fact, it was one of the first orally bioavailable systemic anti-mycotic drugs but is also used locally. Chemically it belongs to the first-generation imidazole anti-fungal drugs (in contrast to triazols). Due to its hepatotoxic properties, oral formulations of Ketoconazole are no longer marketed as anti-fungal medicines as recommended by the European Medicines Agency (EMA) after the Committee for Medicinal Products for Human Use (CHMP) concluded that the risk of liver injury is greater than the benefits in treating fungal infections [60]. In fungi it mechanistically acts as an ergosterole synthesis inhibitor by blocking various CYP-dependent enzymes, thereby explaining both, its hepatotoxic side-effects and its efficacy in Cushing’s treatment: Steroidogenesis in the *zona fasciculata* of the adrenal cortex mainly depends on CYP enzymes [61]. At lower doses, C17-20-lyase and 17-α-hydroxylase are inhibited in both the testicular and adrenal glands. In contrast, cholesterol side-chain cleavage enzymes and 11-β-hydroxylase are inhibited only at higher doses. However, a negative benefit-risk-ratio for one indication (anti-fungal) where alternatives exist does not imperatively mean this also accounts for other indications (Cushing). EMAs safety concern was due to severe hepatic injury but the incidence was only 1 in 15000 cases [62], thus, benefit risk might be different in a disease with high mortality and less alternatives compared to the anti-fungal indication. Currently, Ketoconazole is used off-label in CS, however, marketing authorization applications (MAAs) are underway to change this unsatisfactory status. Thus, future will tell whether Ketoconazole has a positive benefit-risk ratio in CS and currently no final judgment can be made. Ketoconazole may have clinical benefit in CS patients with hyperandrogenic symptoms as it also inhibits steroidogenesis of androgens (for instance in the testes). The occurrence of hirsutism with Metyrapone treatment in women may lead a clinician to choose Ketoconazole in this case or may lead to a combination therapy of Metyrapone and Ketoconazole. Some patients even may require combination of both to achieve control of CS. The dose of Ketoconazole used as monotherapy in patients with CD ranges from 200 mg to 1200 mg per day [63].

### Metyrapone

Metyrapone (Metopirone®) is only registered in four European countries (France, United Kingdom, the Netherlands, and Ireland) through national procedures. From the chemical structure, Metyrapone is a very simple molecule displaying no stereochemistry (2-methyl-1,2-di(pyridin-3-yl)propan-1-one). The drug is very old as it was introduced into the pharmacological armamentarium in the late 50ties of the last century. Thus, in most countries, it was not licensed in a modern way based on careful assessment of quality, safety, and efficacy, but registered and this is the reason why evidence whether Metyrapone is a worthy option in the treatment of CS is rather low. Most publications on the clinical use of Metyrapone are case studies or case series, eventually very small, non-controlled studies. Thus, licensing status is very different among ICH (International Conference on Harmonisation of Technical Requirements for Registration of Pharmaceuticals for Human Use) countries. Metyrapone is more frequently used in the UK in Ireland, while it is not even licensed in Germany, Italy or Spain (to name just a few). Metyrapone is an inhibitor of 11-β-hydroxylase thereby inhibiting endogenous cortisol synthesis. In return, ACTH synthesis is triggered, and this forms the basis for Metyrapone functional test in diagnosis of a properly functioning hypothalamic-pituitary-adrenal (HPA) axis. However, the substance is not listed in current diagnosis guidelines for CS. According to current medicinal guidelines, diagnosis of hypercortisolism (irrespective of its origin) is performed according to the following scheme:

1. Complete blood count including serum electrolytes, blood sugar etc.

2. UFC from 24 h-urine sampling and circadian profile of plasma cortisol, plasma ACTH, dehydroepiandrosterone, testosterone itself, and urine steroid profile

3. Low-Dose-Dexamethasone-Test, High-Dose-Dexamethasone-Test

4. After endocrine diagnostic tests: magnetic resonance imaging (MRI), ultra-sound, computer tomography (CT) and other localization diagnostics

This diagnosis flow-scheme also is reflected in current international text books [64] and for instance in the clinical practice guidelines of the endocrine society [65]:

“*After excluding exogenous glucocorticoid use, we recommend testing for Cushing’s syndrome in patients with multiple and progressive features compatible with the syndrome, particularly those with a high discriminatory value, and patients with adrenal incidentaloma. We recommend initial use of one test with high diagnostic accuracy (urine cortisol, late night salivary cortisol, 1 mg overnight or 2 mg 48-h dexamethasone suppression test). We recommend that patients with an abnormal result see an endocrinologist and undergo a second test, either one of the above or, in some cases, a serum midnight cortisol or dexamethasone-CRH test*.”

Metyrapone, thus, is an excellent example for national traditions determining clinical management of rare diseases. In CS treatment usually is a case-by-case decision because of the heterogeneity of the patients’ population especially in regard to their co-morbidities. Consequently, no (national or international) medicinal guidelines or treatment algorithms of high evidence for management of CS exist. A consensus statement on ACTH-dependent CS published in 2008 by Biller et al. emphasized clinical management of CS as a multi-disciplinary approach on an individualized basis [66].

Despite its efficacy in lowering cortisol blood levels, Metyrapone is not constantly effective and therefore does not cover the entire CS-population. Clinical and/or biochemical improvements can be achieved in 60 to 80% of patients and are usually associated with improvements in symptoms. In general, Metyrapone appeared to be well tolerated, but may sometimes lead to an accumulation of androgens in women during long-term treatment (hirsutism).

### Mifepristone

Mifepristone (experimental name RU-486) originally was developed as the so-called “abortion pill”. Chemically it is an all-trans steroid substituted at positions 11 and 17. Mechanistically, it is an antagonist at the progesterone receptor (PR), thereby explaining its acute abortive effect. It also shows antagonistic activity at the glucocorticoid receptor (GR) with a higher affinity than Dexamethasone. Thus, unlike other agents, Mifepristone does not decrease cortisol synthesis but directly antagonizes its effects. Therefore, it is intuitive that patients dosed with Mifepristone can hardly be diagnosed by serum cortisol measurements as they remain unchanged. Mifepristone is not approved in the indication CS. Side effects include decreased plasma potassium levels with a potential for heart conduction abnormalities, vaginal bleeding, and endometrial thickening (not surprising for a progesterone antagonist).

#### Notions on Mifepristone

Apart from the fact that everyone knows it as an *abortion pill*, it must not be forgotten that Mifepristone originally was developed as an anti-glucocorticoid. Soon afterwards, it was found to block PRs intrinsic activity. Due to its pronounced cortisol-blocking effect, new generation antiprogestins were developed aiming at reducing anti-glucocorticoid activity while maintaining anti-progesterone activity (i.e. ORG-31710, CDB-2914, CDB-4124). However, only Mifepristone has been licensed by US Food and Drug Administration (FDA) to terminate early pregnancy (working as an antiprogestin) or ameliorating the hyperglycemia in CS-patients [67]. In contrast, other PR antagonists like ulipristal and proellex are currently investigated for their potential to alleviate symptoms in endometriosis and uterine fibroids, thereby approaching indications like other hormone ablative medicines (i.e. GnRH analogues) [68].

Interestingly, Tieszen et al. investigated the potential of PR antagonists, to inhibit the growth of cells from endocrine related cancers (i.e. ovarian-, breast-, and prostate cancer cells), expressing different sets of hormone receptors. The authors found, that all cancer cells were inhibited in growth irrespective of their PR status, as there are: MCF-7 breast cancer cells carrying PR, MDA-MB- 231 breast cancer cells with no PR expression, PR negative and androgen receptor positive LNCaP prostate cancer cells, and PR negative androgen receptor positive PC3 prostate cancer cells are all inhibited by mifepristone with similar potency [69].

However, all cell lines under investigation in this study express GR and derivatives of mifepristone with lower GR affinity are less effective. Therefore, it can be concluded that GR action might contribute to anti-tumor effects of PR antagonists. Thus, PRs may not be required for the inhibition of cancer growth triggered by Mifepristone. In addition, it has been shown by another group, that Mifepristone blocked the growth of estrogen receptor negative and PR negative MDA-MB-231 breast cancer cells [70].

### Mitotane

Mitotane is another unusual drug used in certain very rare cases of CS. Mitotane (Lysodren®) is authorized only for symptomatic treatment of advanced (very rare) adrenal cortical carcinoma (ACC). Chemically it is an ortho-derivative of the long-term known insecticide DDT (dichloro-diphenyl-trichloroethane), the bis-trans-form of the molecule. Its biochemical mechanism is not yet fully understood, but inhibition of side-chain cleavage of cholesterol (a decisive step in endogenous steroidogenesis) seems to play a role as well as blockade of 3-β-hydroxysteroid-deshydrogenase [71]. Like its mother substance DDT Mitotane also accumulates in adipose tissue, which might then be entailed with longer half-life. Especially in Cushing’s patients this is of particular concern, as these patients usually present with higher fat mass compared to age-adjusted matched population. Therefore, it usually takes weeks until complete efficacy is reached. In the current SmPC it is even mentioned that 3 - 5 months can be assumed until the intended plasma level of 14 - 20 mg/L is reached. This window should be reached (titrated) by monitoring patients’ blood levels. Thus, Mitotane fulfills the requirements of a *Narrow Therapeutic Index Drug* (NTID) according to the FDA definition. Mitotane has an orphan designation and, thus, the pivotal registration trial was performed in 177 patients only showing an increase in recurrence-free interval after radical surgery followed by Mitotane compared to surgery alone [72]. Grade 3 side effects were mainly neurologic (confusion, ataxia, vertigo) or biochemical (elevated γ-glutamyltransferase). Thus, Mitotane might be of clinical benefit in some patients, adjuvant to radical surgery.

### Pasireotide and somatostatin analogues

Pasireotide mechanistically is a somatostatin-analogue (SSA). SSAs are the first-line medical treatment in GH-secreting adenomas (with the clinical manifestation of acromegaly). Currently two different molecular entities, octreotide (Sandostatin®) and lanreotide (Somatuline®), are marketed. Octreotide, which was licensed 25 years ago, is available as a short-acting subcutaneous formulation for twice-daily administration, and a long-acting (LAR) microsphere preparation administered by intra muscular injection every 4 weeks. Lanreotide is available in a microsphere formulation (sustained release) and a high-concentrate aqueous solution (Autogel). From clinical safety point of view SSA side effects include gallstones and gastrointestinal affection (diarrhea, nausea, abdominal pain). According to the route of administration injection site reactions (pain, swelling) have also been reported.

Pasireotide (experimental name SOM230) itself is a cyclic hexapeptide and was licensed via the central route in Europe in 2012 as first-line pharmacological therapy option. The European Public Assessment Report (EPAR) is dated June 1^st^ 2012 [73].

Endogenous somatostatin is a peptide hormone widely distributed in the endocrine system. Somatostatin action is mediated through five different SSTR subtypes (SSTR-1-SSTR-5). SSTRs belong to the superfamily of tripartite G-protein coupled receptors. They are expressed over the whole body in various tissues, with cells from different tissues expressing different receptor subtypes at different densities. The physiological actions of somatostatin are numerous. It is an inhibitory protein of endocrine secretion of various organs, including the pituitary, pancreas, gastrointestinal tract, thyroid, kidney, and adrenal glands. Among others, it inhibits gallbladder contractility and bile flow, and stimulates gastrointestinal water and electrolyte absorption. Pasireotide has a slightly different SSTR binding profile than the first-generation SSAs Octreotide and Lanreotide, with high affinity to four of the five receptors (SSTR-1, −2, −3 and −5). Compared to Octreotide the binding affinity of Pasireotide is 30 - 40 times greater for SSTR-1 and SSTR-5 and 5 times greater for SSTR-3, whereas the affinity for SSTR-2 is comparable.

In general, medicines for the treatment of CS (i.e. Mifepristone, Ketoconazole) are not selective and initially were not developed for the treatment of CS. In contrast, Pasireotide exhibits at least a subtype-prevalence for subtype-5 of the SSTR in the pituitary gland. SSTR-5 prevalently is expressed at the surface of ACTH secreting cells, thus showing at least partial specificity in lowering excessive cortisol blood levels. Therefore, first-line pharmacological therapy is Pasireotide injection (usually while definitive therapy is awaited).

In addition, the basis of the centralized approval for Pasireotide was a clinical development program consisting (only regarding patients) of

1. a 22-patient phase II study reviewed in [74]

2. a randomized-dose, double-blinded pivotal phase III study [75].

The study design was as follows:

162 patients with Cushing’s disease were randomized (double-blind) to pasireotide 600 μg (n = 82) or 900 μg (n = 80) sc bid. After 3mo, patients with UFC > 2 × ULN (ULN: 145 nmol/24 h) or UFC > baseline were unblinded and the dose increased by 300 μg bid. All others continued on the same double-blind dose to 6mo. Months 6–12 were open-label with dose titration performed when needed. Primary endpoint: UFC ≤ ULN at 6mo without dose up-titration from the randomized dose.

However, it should be noticed that Pasireotide is indicated only in a subgroup of CD-patients and shows satisfactory efficacy only in ~ 30% of patients. Side effects with this agent are similar to those of other SSAs. Hyperglycemia-associated side-effects are of particular concern in CS patients and were reported in 73% of patients in the pivotal study.

### Temozolomide

Temozolomide (Temodal®) is licensed for Grade III and IV glioblastoma multiforme concomitant to radiotherapy [76]. Temozolomide is an orally bioavailable, centrally active alkylating agent. Following a systematic review from 2012 up to now, 46 cases of adenohypophysial tumors are published in Medline that were treated with Temozolomide (30 adenomas, 16 carcinomas). 60% of the adenomas and 69% of carcinomas responded favorably to treatment [77]. Thus, in (rare) cases of highly aggressive growing macroademomas or pituitary carcinomas Temozolomide might be an option.

### Trilostane (only in veterinary use)

Chemically Trilostane is a 2-cyano-4,5-epoxy-steroid. Trilostane reversibly suppresses adrenal function. From its molecular mechanism of action it inhibits cortisol biosynthesis by prevalently blocking 3-β-hydroxysteroide-dehydrogenase. This, however, does not only block cortisol- and corticosteron-synthesis, but also biosynthesis of aldosterone. Thus, Trilostane un-specifically blocks both, glucocorticoids and mineralocorticoids. High doses are reported to even block gonadal steroidogenesis. However, Trilostane is not used as human medicine; its only indication is CS in dogs, irrespective whether CS derives from pituitary or adrenal cortisol-overproduction. For many years before Mitotane has been considered the medical treatment of choice for dogs with adrenal-dependent hyperadrenocorticism [78].

### Therapy of Cushing’s syndrome in children

First of all, in the pediatric population no final conclusion can be drawn on any of the medicines under investigation due to lack of pivotal evidence. This is best reflected by the fact that the EMA accepted a waiver for clinical data from pediatric patients for centrally approved Pasireotide (a so-called “PIP-Waiver”; PIP, pediatric investigation plan). Throughout the SmPCs it is mentioned that only limited evidence is available for children and, thus, the respective medicine is not recommended for use in children. However, in those drugs used off-label even this is based on other indications. There is only one exemption from the rule; for Metyrapone the following can be read in the SmPC: “In children the dosage should be 15 mg/kg bodyweight, with a minimum dose of 250 mg every 4 hours for 6 doses.” However, even for Metyrapone evidence in children is very low (15 documented cases) [2],[79],[80].

### Co-medication for symptomatic therapy

Clinical management of co-morbidities of CS is a complex challenge. Post-surgery management of CS patients is tri-phasic. Ragnarsson and Johannsson summarized systemic therapy while awaiting success of surgery (or radiotherapy) [3].

Usually supportive therapy of CS co-morbidities is by individual therapy depending on clinical presentation of the patient. Certain hazardous (life-threatening) symptoms are treated on a case-by-case decision according to medicinal guidelines of the respective symptoms. The following medicinal guidelines may serve as examples for clinical management of most hazardous co-morbidities:

 ➢ Therapy of **type 2 diabetes mellitus**[81] - evidence level S3

 ➢ Prophylaxis, diagnosis, and treatment of **osteoporosis**[82] - evidence level S3

 ➢ **Depressive disorder**[83] - evidence level S3

 ➢ **Arterial hypertension**[84] - evidence level S2

## Conclusion

CS can arise from different pathological conditions ranging from benign pituitary adenomas to adrenal carcinomas or ectopic cortisol secretion. Thus, various drugs with different target structures and mechanisms of action are used in systemic therapy of CS comprising somatostatin-analogues, cortisol synthesis inhibitors, receptor antagonists and unusual substances like Temozolomide and Mitotane for certain very rare conditions. Some mechanisms of action are well known (Pasireotide, Ketoconazole) others are not fully understood (Mitotane).These drugs differ in terms of safety profile, route of administration, and indications considerably. In addition, safety profiles differ considerably and become hardly predictable, if combinations are needed due to insufficient clinical control of CS by only one substance. Many of these substances are used off-label and constitute an *ultima ratio* approach after failure of preceding therapy options. Thus, clinical and/or at least epidemiological data will be needed to finally judge on safety and efficacy in regard of a debilitating underlying disease.

### Search strategy

#### Recherché of clinical PK-parameter and special populations

i) SmPCs as published on the heads of medicines agencies (HMA) homepage (http://mri.medagencies.org/Human/) were accessed in March and June 2014. Section 5.2 (Pharmacokinetics) of SmPCs systematically was searched for PK-parameters.

ii) A search in PubMed/Medline was performed in March and June 2014 using the international non-proprietary name (INN) of the respective medicinal product combined with the terms “cushing” and “indication” as well as “cushing” and “pediatric”. PubMed recherché was restricted by searching only in clinical trials.

#### Safety profile assessment

SmPCs of currently marketed medicinal products were accessed (if not licensed for treatment of CS, then in other indication; i.e. Ketoconazole, Etomidate, Temozolomide, and Pioglitazone). If not marketed any more (Aminoglutethimide) the last approved SmPC was used except for Trilostane, were veterinary SmPC of Swissmedic was used. Side-effects were categorized by frequency for preferred terms (PT) of the systems organ class (SOC) system (XX = common, very common; X = rare, uncommon; not known).

## Abbreviations

ACC: Adrenal cortical carcinoma

ACTH: Adreoncorticotropic hormone

AUC: Area under the curve

b.i.d.: Twice daily

CD: Cushing’s disease

CMR: Carcinogenic, mutagenic and toxic for reproductive system

CS: Cushing’s syndrome

CT: Computer tomography

CHMP: Committee for medicinal products for human use

C_max_: Maximal plasma concentration

CRH: Corticotropin-releasing hormone

CYP: Cytochrome P450

D_2_: Dopamine receptor subtype 2

EAS: Ectopic ACTH-producing syndrome

EMA: European medicines agency

EPAR: European public assessment report

FDA: US food and drug administration

GABA-A: γ-aminobutyric acid type A

GR: Glucocorticoid receptor

HMA: Heads of medicines agencies

HPA: Hypothalamic-pituitary-adrenal axis

ICH: International conference on harmonisation of technical requirements for registration of pharmaceuticals for human use

INN: Non-proprietary name

MAA: Marketing authorization application

MRI: Magnetic resonance imaging

NTID: Narrow therapeutic index drug

PIP: Pediatric investigation plan

POMC: Pro-opiomelanocortin

PK: Pharmacokinetics

PR: Progesterone receptor

PT: Preferred terms

q.d.: Every day

SCLC: Small-cell lung cancer

SmPC: Summary of product characteristics

SMR: Standardized mortality ratios

SOC: Systems organ class

SSA: Somatostatin-analogue

SSTR: Somatostatine receptor

t_max_: Time after administration when C_max_ is reached

TSS: Trans-sphenoidal surgery

UFC: Urinary free cortisol

## Competing interests

The authors declare that they have no competing interests.

## Authors’ contributions

All authors filed the manuscript, NE and VP performed a systematic search on clinical PK-parameter. All authors read and approved the final manuscript.
